# Development of multivariable prediction models for institutionalization and mortality in the full spectrum of Alzheimer’s disease

**DOI:** 10.1186/s13195-022-01053-0

**Published:** 2022-08-05

**Authors:** Arenda Mank, Ingrid S. van Maurik, Judith J. M. Rijnhart, Els D. bakker, Vincent Bouteloup, Lisa Le Scouarnec, Charlotte E. Teunissen, Frederik Barkhof, Philip Scheltens, Johannes Berkhof, Wiesje M. van der Flier

**Affiliations:** 1 Alzheimer Center Amsterdam, Neurology, Vrije Universiteit Amsterdam, Amsterdam UMC location VUmc, Amsterdam, The Netherlands; 2 Amsterdam Neuroscience, Neurodegeneration, Amsterdam, The Netherlands; 3 Amsterdam UMC, Vrije Universiteit Amsterdam, Department of Epidemiology and Data Science, Amsterdam Public Health Institute, Amsterdam, The Netherlands; 4 Université Bordeaux, Inserm U1219, Institut de Santé Publique, d’Epidémiologie et de Développement (ISPED), Bordeaux, France; 5 Neurochemistry Laboratory and Biobank, Department of Clinical Chemistry, Amsterdam, The Netherlands Neuroscience, VU University Medical Center Amsterdam, Amsterdam UMC, Amsterdam, The Netherlands; 6 Department of Diagnostic Radiology, VU University Medical Center Amsterdam, Amsterdam UMC, Amsterdam, The Netherlands; 7 Queen Square Institute of Neurology and Centre for Medical Image Computing, University College London, London, UK

**Keywords:** Alzheimer’s disease, Mild cognitive impairment, Subjective cognitive decline, Prognosis, Mortality, Institutionalization

## Abstract

**Background:**

Patients and caregivers express a desire for accurate prognostic information about time to institutionalization and mortality. Previous studies predicting institutionalization and mortality focused on the dementia stage. However, Alzheimer’s disease (AD) is characterized by a long pre-dementia stage. Therefore, we developed prediction models to predict institutionalization and mortality along the AD continuum of cognitively normal to dementia.

**Methods:**

This study included SCD/MCI patients (subjective cognitive decline (SCD) or mild cognitive impairment (MCI)) and patients with AD dementia from the Amsterdam Dementia Cohort. We developed internally and externally validated prediction models with biomarkers and without biomarkers, stratified by dementia status. Determinants were selected using backward selection (*p*<0.10). All models included age and sex. Discriminative performance of the models was assessed with Harrell’s C statistics.

**Results:**

We included n=1418 SCD/MCI patients (*n*=123 died, *n*=74 were institutionalized) and *n*=1179 patients with AD dementia (*n*=413 died, *n*=453 were institutionalized). For both SCD/MCI and dementia stages, the models for institutionalization and mortality included after backward selection clinical characteristics, imaging, and cerebrospinal fluid (CSF) biomarkers. In SCD/MCI, the Harrell’s C-statistics of the models were 0.81 (model without biomarkers: 0.76) for institutionalization and 0.79 (model without biomarker: 0.76) for mortality. In AD-dementia, the Harrell’s C-statistics of the models were 0.68 (model without biomarkers: 0.67) for institutionalization and 0.65 (model without biomarker: 0.65) for mortality. Models based on data from amyloid-positive patients only had similar discrimination.

**Conclusions:**

We constructed prediction models to predict institutionalization and mortality with good accuracy for SCD/MCI patients and moderate accuracy for patients with AD dementia. The developed prediction models can be used to provide patients and their caregivers with prognostic information on time to institutionalization and mortality along the cognitive continuum of AD.

**Supplementary Information:**

The online version contains supplementary material available at 10.1186/s13195-022-01053-0.

## Introduction

Alzheimer’s disease (AD) clinically progresses from cognitively normal to mild cognitive impairment (MCI) and ultimately, dementia [1]. Further disease progression is characterized by more severe cognitive and functional impairment, which leads to increased caregiver burden and eventually necessitates institutionalization. The variation in clinical course of individual patients renders the establishment of an individual prognosis challenging. We previously found that time to institutionalization and mortality are important outcomes from the perspective of patients and their caregivers, in both dementia and pre-dementia stages [2]. Providing patients and caregivers with accurate prognostic information on time to nursing home placement (NHP) and mortality can support further care-related decision making and (health-care) planning.

Previous studies on risk factors for institutionalization and mortality have mainly focused on patients with dementia. Factors that reportedly predict institutionalization are older age [3–5], female sex [4–7], Caucasian background [3, 5], living alone [3, 5, 7], more severe functional and cognitive impairment [4, 7–10], and behavioral and psychological symptoms [3, 8, 10]. Shorter survival time in AD dementia has been associated with older age [5, 11–13], male sex [5, 11–13], higher burden of morbidity [11], and lower functional and cognitive abilities [11–13]. The largest part of the AD disease process takes place before the stage of dementia, but studies on prognosis in terms of institutionalization and mortality in pre-dementia stages are hardly available [14].

Few studies have evaluated AD biomarkers as putative markers of institutionalization and mortality. Two studies suggested that cerebrospinal fluid (CSF) biomarkers are not predictive of institutionalization in AD dementia [15, 16]. By contrast, one study observes that extremely high levels of CSF total tau (t-tau) are associated with a higher risk of institutionalization in patients with MCI due to AD [17]. When it comes to mortality, low levels of CSF amyloid-ß_1-42_ (Aβ42) [15, 18] and high levels of phosphorylated tau (p-tau) [12, 18, 19] and t-tau [18, 19] have been found to be associated with an increased risk of mortality in AD dementia. Other studies have shown that white matter hyperintensities (WMH) [20] and global and hippocampal atrophy [12, 21] on magnetic resonance imaging (MRI) were determinants of mortality in AD dementia.

Biomarkers enable the diagnosis of AD before the stage of dementia. However, since curative treatments are not yet available, an early diagnosis of AD may result in more uncertainty for patients and raise questions regarding the disease prognosis. One of these questions pertains to the amount of time left till institutionalization and death. Therefore, it is relevant to develop prognostic models that can provide patients in different stages of the disease information on their prognosis. In the current study, we aimed to predict institutionalization and mortality, based on demographic, clinical, and AD biomarkers (MRI and CSF) information models, in patients with subjective cognitive decline (SCD), mild cognitive impairment (MCI), and AD dementia.

## Methods

### Patients

We included *n*=2597 patients from the Amsterdam Dementia Cohort [22, 23]. All patients had their baseline visit between 2009 and 2020. Inclusion criteria were (1) a baseline diagnosis of Alzheimer’s disease dementia (AD, *n*=1179), mild cognitive impairment (MCI, *n*=582), or subjective cognitive decline (SCD, *n*=836) and (2) availability of baseline MRI and/or CSF biomarkers. Mean follow-up was 3.8±2.9 years for institutionalization and 4.7±2.9 years for mortality in SCD/MCI patients (SCD and MCI). In AD dementia, mean follow-up was 2.9±2.4 years for institutionalization and 4.2±2.4 years for mortality.

All patients received a standardized dementia diagnostic work-up, which consisted of medical history; neurological, physical and neuropsychological evaluation; MRI; laboratory tests; and lumbar puncture for CSF measurement [22, 23]. Subsequently, clinical diagnosis (i.e., SCD, MCI, or AD dementia) was made in a multi-disciplinary meeting. Patients were diagnosed with AD dementia or MCI according to the National Institute on Aging-Alzheimer’s Association (NIA-AA) criteria [24, 25]. Patients were labeled SCD when they presented with cognitive complaints, had normal clinical and cognitive test results, and did not meet the criteria for MCI, dementia, or other neurologic or psychiatric conditions [26].

We obtained written informed consent from all patients. The study was approved by the Medical Ethics Review Committee of the VU University Medical Center. This study is reported in accordance with the Transparent Reporting of a multivariable prediction model for Individual Prognosis Or Diagnosis (TRIPOD) guideline [27].

### Candidate predictors

Variables considered for the model were age, sex, Mini-Mental State Examination (MMSE) [28], Charlson Comorbidity Index (CCI), Neuropsychiatric Inventory (NPI) [29], APOE e4 status, MRI medial temporal lobe atrophy (MTA), global cortical atrophy (GCA), white matter hyperintensities (WMH), CSF Aβ42 and CSF p-tau. All candidate predictors were measured at the first recorded diagnosis in the memory clinic. Comorbidity was defined using CCI, which was calculated based on medical history and medication use [30]. As part of the CCI, we used renal function (MDRD; Modified Diet in Renal Disease study) to score for the presence of moderate to chronic kidney disease [31, 32].

### MRI acquisition

MRI scans were available in *n*=2074 (80%) patients. MRI of the brain was performed on 1.0 or 1.5 Tesla (T) MRIs (Siemens Magnetom Avanto, Vision, Impact and Sonata, GE Healthcare Signa HDXT) and after 2008 on 3T MRI (MR750, GE Medical Systems, Milwaukee, WI, USA; Ingenuity TF PET/MR, Philips Medical Systems, Best, The Netherlands; Titan, Toshiba Medical Systems, Japan). MRI scans were performed according to a standardize protocol and reviewed by experienced neuroradiologists. Visual rating scales were used to rate atrophy: Medial temporal lobe atrophy (MTA, 0–4) [33] and Global cortical atrophy (GCA, 0–3) [34]. White matter hyperintensities (WMH) were rated with the Fazekas scale (0–3) [35].

### CSF analysis

CSF was available in *n*=2057 (79%) patients. CSF was obtained by lumbar puncture, collected in polypropylene tubes (Sarstedt Nurnberg, Germany), and processed according to international guidelines [36]. Before 2018, amyloid beta (Aβ42), total tau (t-tau), and phosphorylated threonine 181 (p-tau) were measured using sandwich ELISA’s (Innotest, Fujirebio, Gent, Belgium) (*n*=1653) [37]. Amyloid beta values were drift corrected [38]. After 2018, CSF was analyzed using Elecsys (*n*=404). All CSF values were transformed to Elecsys values using a previously published bridging equation [39].

### Amyloid status

We performed a subgroup analysis in the subgroup of amyloid-positive patients only. We used amyloid-PET and CSF Aβ42 to determine whether a patient was amyloid-positive or amyloid-negative. Patients were categorized in the amyloid-positive group if they had a positive amyloid-PET scan and/or CSF Aβ42<1000 pg/ml. Amyloid-PET scans were performed using 3-Tesla Philips Ingenuity TF PET/MR, Philips Ingenuity TF PET/CT, and Philips Gemini TF PET/CT scanners. PET scans were visually rated as positive or negative by a trained nuclear medical physician. The amyloid PET procedure using ^18^F-florbetaben, ^18^F-Florbetapir, ^18^F-flutemetamol, or ^11^C-Pittsburgh compound B (PiB) has been described in detail elsewhere [40].

### Institutionalization and mortality

Data on institutionalization and mortality were derived from administrative data sources made available by Statistic Netherlands (in Dutch: Centraal Bureau voor de Statistiek) [41]. Statistics Netherlands collects data on every registered person in the Netherlands. All included patients of the ADC cohort were linked with the Statistic Netherlands data based on the unique combination of date of birth, sex, postal code, and house number. Institutionalization was defined as permanent admission to a nursing home. Mortality was defined as all-cause mortality. Date on admission to a nursing home originated from the Dutch long-term care insurance scheme and the date of death from the municipal personal records database. Time to institutionalization and time to death were measured in years from the first recorded diagnosis in the memory clinic to the date of institutionalization (between 1 January 2009 and 31 December 2019) and the date of death (between 1 January 2009 and 31 December 2020).

### Statistical analyses

All analyses were performed in STATA SE version 16.0. We constructed univariable and multivariable cox regression models, stratified by dementia status, to predict 1) institutionalization and 2) all-cause mortality. In the multivariable models, we first entered age and sex, and then selected other candidate variables using the backward selection procedure if *p*-value <0.10. Missing data on candidate predictors (NPI, MTA, GCA, WMH, CSF Aβ42 and CSF p-tau) were imputed via Multiple Imputation using Chained Equations (MICE) (Additional file 1) [42]. Model 1 is an univariable model adjusted for age and sex. Model 2 is a multivariable model consisting of a model built with all candidate predictors and backward selection. In addition, we developed two submodels of model 2; a model without CSF biomarkers (model 2a) and a model without MRI and CSF biomarkers (model 2b). Discriminative performance of the multivariable models was assessed with Harrell’s C statistics [43]. Accuracy of the models was assessed with the 3-year Brier scores [44, 45]. In an additional analysis, we performed a subgroup analysis, restricted to amyloid-positive patients (i.e., AD continuum).

Fivefold cross-validation was performed to evaluate the models. Data was randomly split in 80% for training and 20% for testing. Each time, models were constructed from the training set using backward selection. Harrell’s C and hazard ratios were calculated on the test set. Subsequently, we calculated the average of the hazard ratios and Harrell’s C-statistics of the five different models and compared this with the hazard ratios and Harrell’s C-statistics of the main models.

The concordance between the predicted and observed outcomes was assessed by comparing the probabilities of institutionalization and mortality for each time point as estimated by the Cox model to those obtained by the Kaplan-Meier method, respectively [46]. For this purpose, the prognostic index was calculated based on the models (model 2; constructed with all candidate predictors) for each test set in the five-fold cross-validation based on the linear predictor from the corresponding training set and we categorized the prognostic index into four risk groups: good prognosis (<16th percentile), fairly good prognosis (16–50th percentile), fairly poor prognosis (50–84th percentile), and poor prognosis (>84th percentile). For each of these groups, we compared the average survival curve based on Cox regression with the Kaplan-Meier curve. The proportional hazards assumption was evaluated by comparing the Kaplan-Meier curves across the four risk groups. Crossing curves indicate that the proportional hazards assumption is classified as violated.

For the model predicting institutionalization in AD dementia, the curves showed that this model discriminates well between subjects and that calibration is good for the first 6 years of follow-up and weaker after 6 years of follow-up. Therefore, for this model, we evaluated the proportional hazard assumption of each covariate within the Cox model using the Schoenfeld residuals [47]. For the variables with a statistically significant Schoenfeld residuals test, we additionally estimated time-varying coefficients to demonstrate how the prognostic power of the significant variables of the Schoenfeld residuals changes over time.

### External validation

External validation was performed in the Memento cohort [48]. This is a multi-center cohort of participants consulting French memory clinics and presenting with cognitive complaints or MCI. At memory clinics, baseline data collection included demographic, social, clinical data, neuroimaging (MRI, FDG PET), and fluid (blood, CSF). The brain parenchymal fraction (white plus grey matter volumes divided by intracranial volume) was used as a measure for GCA and then standardized (converted to a z-score) in the Cox models. CSF biomarkers were obtained from Fuijrebio kits and then converted to Elecsys. We fitted the regression coefficients from the ADC model in Memento and evaluated the resulting Harrell’s C.

## Results

### AD dementia vs. SCD/MCI patients

We included *n*=1418 SCD/MCI patients and *n*=1179 patients with AD dementia. Baseline characteristics are shown in Table 1. Mean age of SCD/MCI patients was 63±7 years, *n*=532 (38%) were female, and the mean MMSE score was 27±2. AD dementia patients were 65±7 years, *n*=624 (53%) were female and the MMSE score was 20±5. In SCD/MCI patients, *n*=123 (9%) died and *n*=74 (5%) were institutionalized. In AD dementia, *n*=413 (35%) patients died and *n*=453 (38%) patients were institutionalized.

**Table 1:** Baseline characteristics

	SCD/MCI (***n***=1418)	AD dementia (***n***=1179)	***p***-value
**Age**	63±7	65±7	<0.001
**Sex, female**	532 (38%)	624 (53%)	<0.001
**MMSE**	27±2	20±5	<0.001
**Diagnoses**			
**SCD**	836 (59%)		
**MCI**	582 (41%)		
**NPI**	10±9	12±9	<0.001
**CCI**	2.5±1.4	3.6±1.3	<0.001
**APOE e4 carrier**	618 (58%)	751 (53%)	<0.001
**MRI**			
**GCA**	0.5±0.6	1.1±0.6	<0.001
**MTA**	0.6±0.6	1.4±0.7	<0.001
**WMH**	0.9±0.8	1.0±0.7	<0.001
**CSF, pg/ml**			
**Aβ42**	1394±498	749±274	<0.001
**p-tau**	21±12	35±16	<0.001

Data is represented as mean±SD, median (range) or *n* (%)

*AD* Alzheimer’s disease, *SCD* subjective cognitive decline, MCI= mild cognitive impairment, *MMSE* Mini-Mental State Examination, *NPI* Neuropsychiatric Inventory, *CCI* Charlson Comorbidity Index, *MRI* magnetic resonance imaging, *GCA* global cortical atrophy (0–3), *MTA* medial temporal lobe atrophy (0–4), *WMH* white matter hyperintensities (0–3), *CSF* cerebrospinal fluid, *Aβ42* β-Amyloid 1–42, *p-tau* Tau phosphorylated at threonine 181. CSF values were bridged to Elecsys value

Univariable analysis (Tables 2 and 3) shows that older age, lower MMSE, higher NPI scores, higher burden of morbidity (CCI), more abnormal MRI scores (GCA and MTA), lower CSF Aβ42, and higher CSF p-tau increased the risk of both mortality and institutionalization in both strata. Of note, more severe WMH significantly increased the risk of institutionalization in SCD/MCI patients but decreased the risk of institutionalization in AD dementia.

**Table 2:** Univariable and multivariable Cox regression models for the prediction of institutionalization and mortality in SCD/MCI patients

	Institutionalization					Mortality				
	Univariable	Model 1	Model 2			Univariable	Model 1	Model 2		
		Age and sex adjusted		Without CSF	Without CSF/MRI		Age and sex adjusted		Without CSF	Without CSF/MRI
**Age**	1.09* (1.05; 1.13)	1.10* (1.06; 1.14)	1.03 (1.00; 1.07)	1.07 (1.02; 1.11)	1.10 (1.06; 1.14)	1.12* (1.09; 1.16)	1.12* (1.09; 1.15)	1.04 (1.00; 1.07)	1.06 (1.02; 1.09)	1.08 (1.05; 1.12)
**Sex, female**	1.21 (0.73; 2.00)	1.35 (0.81; 2.23)	1.52 (0.98; 2.60)	1.67 (1.00; 2.83)	1.56 (0.92; 2.63)	0.72 (0.49; 1.06)	0.75 (0.51; 1.10)	0.97 (0.64; 1.45)	0.96 (0.65; 1.43)	0.88 (0.59; 1.32)
**MMSE**	0.83* (0.77; 0.90)	0.83* (0.76; 0.91)	0.91 (0.83; 1.00)	0.84 (0.77; 0.91)	0.84 (0.77; 0.92)	0.94 (0.88; 1.01)	0.95 (0.88; 1.02)			
**NPI**	1.03* (1.00; 1.06)	1.03* (1.01; 1.06)	1.03 (1.00; 1.06)		1.03 (1.00; 1.06)	1.03* (1.01; 1.05)	1.02* (1.00; 1.04)	1.02 (1.00; 1.04)		1.02 (1.00; 1.04)
**CCI**	1.22* (1.07; 1.39)	1.00 (0.81; 1.23)				1.50* (1.38; 1.63)	1.29* (1.15; 1.46)	1.27 (1.12; 1.43)	1.23 (1.09; 1.39)	1.28 (1.14; 1.45)
**APOE e4**	1.96* (1.18; 3.27)	1.91* (1.15; 3.19)		1.67 (1.00; 2.79)	1.85 (1.10; 3.09)	1.36 (0.94; 1.96)	1.32 (0.91; 1.90)			
**GCA**	2.74* (1.92; 3.92)	2.21* (1.45; 3.36)	2.08 (1.34; 3.23)	2.08 (1.32; 3.26)		2.93* (2.27; 3.80)	1.89* (1.39; 2.56)	1.39 (0.97; 1.97)	1.46 (1.03; 2.06)	
**MTA**	1.89* (1.34; 2.58)	1.36 (0.91; 2.04)				2.52* (2.04; 3.12)	1.76* (1.37; 2.27)	1.50 (1.11; 2.01)	1.44 (1.08; 1.93)	
**WMH**	1.60* (1.20; 2.13)	1.25 (0.91; 1.71)				1.47* (1.19; 1.82)	1.08 (0.85; 1.37)			
**CSF Aβ**_**42**_^a^	0.85* (0.80; 0.90)	0.87* (0.82; 0.92)	0.90 (0.84; 0.96)			0.92* (0.89; 0.96)	0.95* (0.92; 0.99)	0.96 (0.92; 1.00)		
**CSF p-tau**	1.05* (1.04; 1.06)	1.04* (1.02; 1.05)	1.02 (1.01; 1.04)			1.03* (1.02; 1.04)	1.02* (1.00; 1.03)	1.02 (1.00; 1.03)		
**Harrell’s C**			0.81 (0.76; 0.86)	0.76 (0.71; 0.82)	0.76 (0.70; 0.82)			0.79 (0.75; 0.83)	0.79 (0.75; 0.83)	0.76 (0.71; 0.80)
**3-year Brier score**			0.015 (0.008; 0.021)	0.015 (0.008; 0.021)	0.015 (0.008; 0.021)			0.024 (0.016; 0.032)	0.0244 (0.016; 0.032)	0.024 (0.016; 0.032)

Data is represented as Hazard Ratio (95%CI) and Harrell’s C (95%CI)

We used all variables as continuous variables in the models, except for the dichotomous variables gender and APOE e4

*AD* Alzheimer’s disease, *95%CI* 95% confidence interval, *NPI* Neuropsychiatric Inventory, *MMSE* Mini-Mental State Examination, *CCI* Charlson Comorbidity Index, *GCA* global cortical atrophy, *MTA* medial temporal lobe atrophy, *WMH* white matter hyperintensities, *CSF* cerebrospinal fluid, *Aβ*_*42*_ β-Amyloid 1–42, *p-tau* Tau phosphorylated at threonine 181

^a^Hazard ratio for every 100 pg/ml

*p<0.05 in univariate analysis

**Table 3:** Univariable and multivariable Cox regression models for the prediction of institutionalization and mortality in AD dementia patients

	Institutionalization					Mortality				
	Univariable	Model 1	Model 2			Univariable	Model 1	Model 2		
		Age and sex adjusted		Without CSF	Without CSF/MRI		Age and sex adjusted		Without CSF	Without CSF/MRI
**Age**	1.00 (0.99; 1.01)	1.00 (0.99; 1.01)	1.00 (0.98; 1.01)	1.00 (0.99; 1.02)	1.02 (1.00; 1.04)	1.02* (1.01; 1.04)	1.02* (1.01; 1.04)	1.02 (1.00; 1.03)	1.02 (1.00; 1.03)	1.03 (1.01; 1.04)
**Sex, female**	0.95 (0.79; 1.15)	0.95 (0.79; 1.15)	0.93 (0.77; 1.13)	0.94 (0.78; 1.14)	0.88 (0.73; 1.07)	0.76* (0.63; 0.92)	0.76* (0.63; 0.92)	0.70 (0.57; 0.85)	0.70 (0.57; 0.85)	0.71 (0.58; 0.86)
**MMSE**	0.93* (0.91;0.94)	0.93* (0.91; 0.94)	0.94 (0.92; 0.96)	0.93 (0.92; 0.95)	0.93 (0.91; 0.94)	0.93* (0.92; 0.95)	0.93* (0.91; 0.94)	0.94 (0.92; 0.95)	0.94 (0.92; 0.95)	0.93 (0.91; 0.95)
**NPI**	1.03* (1.02; 1.04)	1.03* (1.02; 1.04)	1.03 (1.02; 1.04)	1.03 (1.02; 1.04)	1.03 (1.02; 1.04)	1.02* (1.01; 1.03)	1.02* (1.01; 1.03)	1.01 (1.00; 1.02)	1.01 (1.00; 1.02)	1.01 (1.00; 1.02)
**CCI**	1.00 (0.93; 1.08)	1.00 (0.91; 1.11)			0.90 (0.81; 1.00)	1.16* (1.08; 1.25)	1.13* (1.02; 1.24)			
**APOE e4**	1.03 (0.84; 1.26)	1.03 (0.84; 1.26)				0.93 (0.76; 1.15)	0.94 (0.77; 1.16)			
**GCA**	1.26* (1.07; 1.48)	1.28* (1.08; 1.50)				1.49* (1.26; 1.76)	1.43* (1.20; 1.69)	1.21 (1.01; 1.45)	1.21 (1.01; 1.45)	
**MTA**	1.37* (1.20; 1.56)	1.43* (1.24; 1.64)	1.34 (1.15; 1.55)	1.30 (1.12; 1.51)		1.39* (1.23; 1.58)	1.32* (1.15; 1.51)			
**WMH**	0.97 (0.85; 1.09)	0.96 (0.84; 1.10)	0.84 (0.75; 0.96)	0.83 (0.72; 0.95)		1.30* (1.14; 1.48)	1.27* (1.10; 1.46)	1.18 (1.02; 1.35)	1.18 (1.02; 1.35)	
**CSF Aβ**_**42**_^a^	1.00 (0.96; 1.03)	0.99 (0.96; 1.03)				0.96* (0.92; 0.99)	0.95* (0.92; 0.99)			
**CSF p-tau**	1.01* (1.00; 1.01)	1.01* (1.00; 1.01)	1.01 (1.00; 1.01)			1.01* (1.00; 1.01)	1.01* (1.00; 1.01)			
**Harrell’s C**			0.68 (0.65; 0.70)	0.68 (0.65; 0.70)	0.67 (0.64; 0.70)			0.65 (0.62; 0.68)	0.65 (0.62; 0.68)	0.65 (0.62; 0.68)
**3-year Brier score**			0.174 (0.161; 0.187)	0.174 (0.161; 0.187)	0.177 (0.164; 0.190)			0.091 (0.077; 0.105)	0.091 (0.077; 0.105)	0.091 (0.077; 0.105)

Data is represented as Hazard Ratio (95%CI) and Harrell’s C (95%CI)

We used all variables as continuous variables in the models, except for the dichotomous variables gender and APOE e4

*AD* Alzheimer’s disease, *95%CI* 95% confidence interval, *NPI* Neuropsychiatric Inventory, *MMSE* Mini-Mental State Examination, *CCI* Charlson Comorbidity Index, *GCA* global cortical atrophy, *MTA* medial temporal lobe atrophy, *WMH* white matter hyperintensities, *CSF* cerebrospinal fluid, *Aβ*_*42*_ β-Amyloid 1–42, *p-tau* Tau phosphorylated at threonine 181

^a^Hazard ratio for every 100pg/ml

*p<0.05 in univariate analysis

Subsequently, we used backward elimination to select determinants for the multivariable model. Tables 2 and 3 show the multivariable models for the prediction of institutionalization and mortality stratified by dementia status. Discriminative performance was higher in SCD/MCI patients than in patients with AD dementia for both institutionalization (Harrell’s C (95%CI): 0.81 (0.76; 0.86) vs 0.68 (0.65; 0.70)) and mortality (Harrell’s C (95%CI): 0.79 (0.75; 0.83) vs 0.65 (0.62; 0.68)).

The model in SCD/MCI patients included clinical characteristics (age, sex, MMSE, NPI) combined with imaging (GCA) and CSF biomarkers (Aβ42 and p-tau) for institutionalization (Harrell’s C=0.81(0.76; 0.86)). Compared to this model, the model for mortality included CCI and MTA, but not MMSE (Harrell’s C (95% CI)=0.79 (0.75; 0.83)). Cross-validation showed a Harrell’s C in the same range for both institutionalization (Harrell’s C (95% CI)=0.76 (0.64–0.87)) and mortality (Harrell’s C (95%)=0.75 (0.66–0.85)) (Additional file 2). Additional models constructed under the assumption that MRI and/or CSF were not available, resulted in somewhat lower Harrell’s C, although Harrell’s C remained within the same range compared to the model built with all candidate predictors (Table 2).

In AD dementia, the model for institutionalization included clinical characteristics (age, sex, MMSE, NPI), imaging (MTA, WMH) and CSF p-tau (Harrell’s C (95%CI)=0.68 (0.65; 0.70)). Compared to this model, the model for mortality included GCA, but not MTA and p-tau (Harrell’s C (95%CI)=0.65 (0.62; 0.68)). Of note, more severe WMH decreased the risk of institutionalization, but increased the risk of mortality in AD patients. Cross-validation showed a Harrell’s C in the same range for both institutionalization (Harrell’s C (95%CI)= 0.66 (0.59–0.72)) and mortality (Harrell’s C (95%CI)=0.63 (0.56–0.70)) (Additional file 3). Models constructed without biomarkers had similar discriminative values compared to the model built with all candidate predictors.

The 3-year Brier scores of the models were 0.015 (0.008; 0.021) for predicting institutionalization in SCD/MCI, 0.024 (0.016; 0.032) for predicting mortality in SCD/MCI, 0.174 (0.161; 0.187) for predicting institutionalization in AD dementia and 0.091 (0.077; 0.105) for predicting mortality in AD dementia (Tables 2 and 3).

**Fig. 1:**
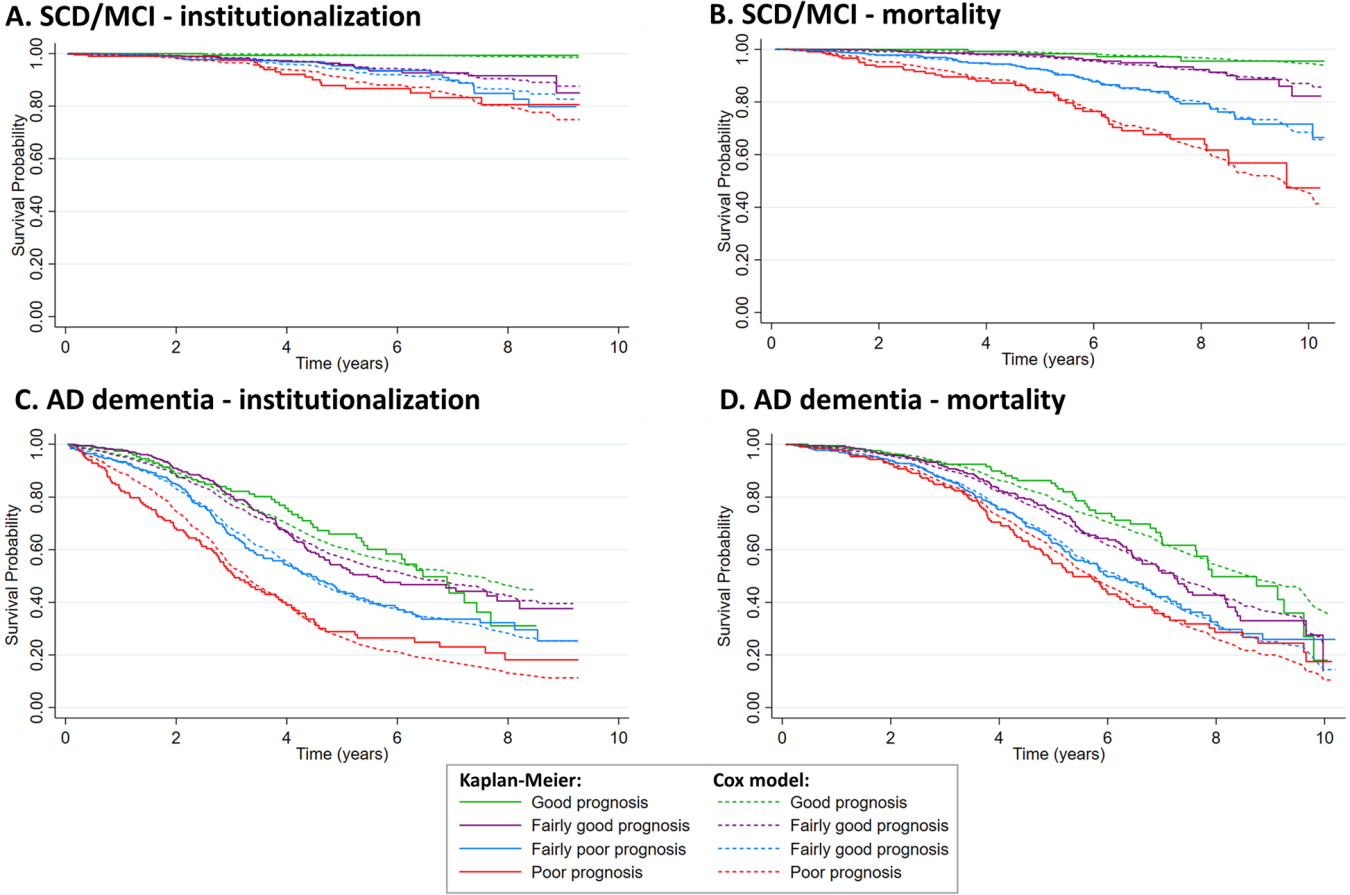
Fitting of the models, stratified by dementia status, to predict institutionalization and mortality. The concordance between the predicted and observed outcomes was assessed by comparing the probabilities of institutionalization and mortality for each time point as estimated by the Cox model to those obtained by the Kaplan-Meier method, respectively. For this purpose, the prognostic index was calculated based on the models (model 2; constructed with all candidate predictors) for each test set in the fivefold cross-validation based on the linear predictor from the corresponding training set and we categorized the prognostic index into four risk groups

Figure 1 shows the comparison between the probabilities estimated by the Cox model and those obtained by the Kaplan-Meier method for each of the four prognosis groups based on the prognostic index. The curves for the prognosis groups were more widely separated in SCD/MCI patients for mortality compared to AD dementia, indicating better discrimination for SCD/MCI patients. SCD/MCI patients generally had similar probabilities obtained by Cox and Kaplan-Meier method, until at least five years of follow-up (Fig. 1A and B). For the fairly poor prognosis group in AD dementia, similar probabilities were obtained by the Cox model and Kaplan-Meier model for institutionalization (Fig. 1C). In the fairly good prognosis group in AD dementia, there is an underestimation for institutionalization in the first four years, while in the poor prognosis group in AD there is an overestimation for institutionalization in the first years. In all four prognosis groups in AD, dementia mortality was accurately predicted by the Cox model up to 3 years follow-up (Fig. 1D).

For the model predicting institutionalization in AD dementia, the curves in Fig. 1 showed that the model discriminates well between subjects and that calibration is good for the first 6 years of follow-up and weaker after 6 years of follow-up (Fig. 1C). The Schoenfeld residuals showed that the proportional hazard assumption was violated for the covariates MMSE and NPI in the model for institutionalization (Additional file 4). To demonstrate how the prognostic power of MMSE and NPI changes over time, we reported time-varying effects of MMSE and NPI on the hazard ratio to Additional file 5. The results indicate that the effect of MMSE and NPI on the hazard ratio is weaker after 6 years of follow-up than in the first 6 years.

### Amyloid-positive patients

In an additional set of analyses, we restricted our multivariable models for the prediction of institutionalization and mortality in amyloid-positive patients (Additional files 6 and 7). There were *n*=372 SCD/MCI and *n*=1039 dementia AD patients, based on positive amyloid PET and/or CSF Aβ42. In SCD/MCI, *n*=44 (12%) died and *n*=39 (10%) were institutionalized. In AD dementia, *n*=350 (34%) patients died and *n*=374 (36%) patients were institutionalized. The model in SCD/MCI patients included clinical characteristics (age, sex, MMSE) combined with imaging (GCA, MTA, WMH) and CSF p-tau for institutionalization (Harrell’s C (95%CI)=0.72 (0.66; 0.84)). Compared to this model, the model for mortality did not include MMSE, GCA, and WMH (Harrell’s C (95%)=0.74 (0.66; 0.83)). In AD dementia patients, the model for institutionalization included clinical characteristics (age, sex, MMSE, NPI) combined with imaging (MTA, WMH) and CSF p-tau (Harrell’s C=0.68 (0.64; 0.71)). Compared to this model, the model for mortality did not include CSF p-tau (Harrell’s C (95% CI)=0.65 (0.62; 0.68)).

### External validation

For external validation, we included *n*=2308 SCD/MCI patients (*n*=1943 MCI, *n*=365 SCD) from the memento cohort (Additional file 8). Of these patients, *n*=95 (4%) died and *n*=66 (3%) were institutionalized. Mean follow-up was 4±2 years for both outcomes. In *n*=404 patients CSF data was available and in *n*=2153 patients MRI data was available. External validation shows similar discrimination for the models to predict institutionalization (Harrell’s C (95%CI)=0.79 (0.65; 0.93)) and mortality (Harrell’s C (95%CI)=0.72 (0.60; 0.85)) in SCD/MCI patients (Additional file 9).

## Discussion

We developed validated models to predict institutionalization and mortality along the AD continuum. Models for institutionalization and mortality included clinical characteristics, imaging and CSF biomarkers in both SCD/MCI patients and AD dementia patients. Discriminative performance was better in SCD/MCI patients than in patients with AD dementia. Models based on amyloid-positive patients only had a similar discriminative performance.

Extending on previous studies on the prediction of institutionalization and mortality in AD, we included biomarkers as pathophysiological indicators of the disease in the models. From earlier studies, we know that these biomarkers are strongly related to cognitive decline over time and the progression from pre-dementia to dementia stages [49]. Abnormal CSF biomarkers were associated with rapid cognitive decline [50], which lead to functional impairment and eventually necessitates institutionalization and increases the risk of death. Furthermore, we not only predicted mortality and institutionalization in AD dementia, but also in patients with SCD and MCI. It is relevant to develop prognostic models that can provide patients with different levels of cognitive impairment information on their prognosis. Whereas most prediction models in the literature focus on dementia as the endpoint [49], patients may be looking for more practical information. In our previous study, patients and caregivers reported the time to institutionalization and mortality as important outcomes [2]. Taking this need as expressed by patients and families as a starting point, in this study, we developed models to predict time to institutionalization and mortality for patients with and without dementia. Information derived from such models can support clinicians and patients in the timing of advance care planning and shared decision-making.

The few available models that predict mortality have been developed for patients with dementia, and they did not contain any information on biomarkers. A former study that predicted survival in dementia, included age, sex, setting of care, and CCI [51]. A study from the Swedish Dementia registry included age, sex, CCI, MMSE, and dementia type in the model [11]. Similar to our study, male sex increased the risk of mortality. Compared to these previous models, we observed a lower discriminative performance in the model for mortality in AD dementia patients. Possible explanations for the difference in the discriminative performance are our models are based on a smaller cohort, a younger population, and a lower burden of morbidity. In contrast with the previously reported models, CCI as a measure of comorbidity was not retained in our model, perhaps due to the relatively young age of our sample. However, CCI added predictive value in the models predicting survival in SCD/MCI patients. A potential explanation for this finding is that the SCD/MCI patients who died during follow-up had a cause of death other than Alzheimer’s disease.

Most previous studies on predictors for institutionalization have been performed in patients with dementia, contain no biomarkers, and did not assess discrimination. One study in patients with dementia showed that living alone, dependency in activities of daily living (ADL), lower MMSE, higher NPI score, and black/Hispanic ethnicity increased the risk of institutionalization [52]. Our model for AD dementia also includes MMSE and NPI and shows similar discriminative performance. Another study showed that MMSE, NPI, instrumental ADL, and non-spousal informal caregiver were determinants of time to institutionalization [53]. This study has not assessed the discrimination of the models. We add biomarkers to the institutionalization models and evaluated the discrimination of models in dementia and pre-dementia stages.

### Strengths and limitations

A strength of our study is that we included a large sample of patients with diagnoses ranging from SCD, MCI to AD dementia. All SCD/MCI patients visited the memory clinic, being worried about their cognitive complaints. Prognostic information about mortality and institutionalization is also relevant to them. However, since we intended to provide prognostic information along the AD continuum and not all patients with SCD or MCI had underlying AD. We performed an additional analysis restricted to amyloid-positive patients only. The resulting model was very comparable in terms of determinants and prognostic performance. Another strength is that we evaluated three different models with and without biomarkers, using fivefold cross-validation and we validated the models externally. We observed good discriminative performance, indicating that the models are robust and may have value in clinical practice.

A limitation of this study is that many variables are included in the models. In practice, information may not always be available on all these variables for each patient. Therefore, we constructed additional models under the assumption that CSF and/or MRI were not available. These models had approximately the same discriminative performance. Therefore, mortality and institutionalization can still be accurately predicted in the absence of biomarker information. However, when MRI and/or CSF were available then the inclusion of this information in the model improves the accuracy of the prediction of mortality and institutionalization. Another potential limitation is that only a small percentage of patients were institutionalized or died in the group with SCD/MCI patients. This may explain the higher discriminative performance in the models for SCD/MCI patients, and further illustrates the need for truly longitudinal data to derive meaningful models in this highly relevant population. Another limitation is the violation of the proportional hazards assumption after 6 years of follow-up in the model predicting institutionalization for the good and fairly good prognosis groups in AD patients. The Schoenfeld residuals showed that the proportional hazards assumption is violated for MMSE and NPI in the model predicting institutionalization in AD dementia. This suggests that MMSE and NPI measured at the time of diagnosis lose their predictive power over time, probably because of disease progression. Therefore, we recommend to update the cognitive tests regularly in the model for accurate long-term prediction. Finally, the statistical power is too low to do a subgroup analysis based on the cause of death. However, the classification of AD-related mortality is a complex procedure, because patients with AD die due to the complications of dementia such as dehydration, malnutrition, or infection. For example, swallowing problems in an AD patient can lead to aspiration pneumonia which results in death, or an AD patient with cancer as comorbidity forgets to visit a doctor and subsequently dies as a result of advanced-stage cancer [54]. Therefore, it is more relevant to predict all-cause mortality instead of AD-related mortality.

## Conclusion

In conclusion, we constructed prediction models to predict institutionalization and mortality in patients with diagnoses ranging from SCD and MCI to AD dementia. The model for institutionalization and mortality included clinical characteristics, imaging, and CSF biomarkers for both SCD/MCI and AD dementia patients. The models can be used to provide patients in both pre-dementia and dementia stages and their caregivers’ prognostic information on the time to institutionalization and mortality.

## Supplementary Information

**Additional file 1.** Multiple Imputation using Chained Equations (MICE).

**Additional file 2.** Five-fold cross-validation of the prediction models in SCD/MCI patients.

**Additional file 3.** Five-fold cross-validation of the prediction models in AD dementia.

**Additional file 4.** Schoenfeld residuals for the model (model 2) predicting institutionalization in AD dementia.

**Additional file 5.** Time-varying hazard ratios of MMSE and NPI after six years of follow-up for the model (model 2) predicting institutionalization in AD dementia.

**Additional file 6.** Univariable and multivariable cox regression models for the prediction of institutionalization and mortality in SCD/MCI amyloid-positive patients.

**Additional file 7.** Univariable and multivariable cox regression models for the prediction of institutionalization and mortality in amyloid-positive patients with AD dementia.

**Additional file 8.** Baseline characteristics Memento cohort.

**Additional file 9.** Harrell’s C Memento cohort based on regression variables of the Amsterdam Dementia Cohort (ADC) models.

## Data Availability

The datasets used and/or analyzed during the present study are available from the corresponding author on reasonable request.

## Abbreviations

Aβ42: β-Amyloid 1–42
p-tau: Tau phosphorylated at threonine 181
AD: Alzheimer’s disease
95%CI: 95% confidence interval
APOE: Apolipoprotein E
NPI: Neuropsychiatric Inventory
MMSE: Mini-Mental State Examination
CCI: Charlson Comorbidity Index
MRI: Magnetic resonance imaging
GCA: Global cortical atrophy
MTA: Medial temporal lobe atrophy
WMH: White matter hyperintensities
CSF: Cerebrospinal fluid
