# Supplementary material for: Development of multivariable prediction models for institutionalization and mortality in the full spectrum of Alzheimer’s disease
Source: Alzheimers Res Ther. 2022 Aug 5;14:110. doi: 10.1186/s13195-022-01053-0 (PMC9354423; doi:10.1186/s13195-022-01053-0)
Supplement: Supplementary file 1 — Additional file 1. Multiple Imputation using Chained Equations (MICE). [file 13195_2022_1053_MOESM1_ESM.docx]

**Additional file 1**. **Multiple Imputation using Chained Equations (MICE)**

Missing data on candidate predictors (NPI, MTA, GCA, WMH, CSF Aβ42 and CSF p-tau) were imputed via Multiple Imputation using Chained Equations (MICE).^1^ In SCD/MCI patients, the number of missing values were n=364(26%) NPI, n=223(16%) MTA, n=222(16%) GCA, n=219(15%) WMH, n=339(24%) CSF Aβ42 and n=340(24%) CSF p-tau. In AD dementia, the n(%) missing values were n=155(13%) NPI, n=297(25%) MTA, n=301(26%) GCA, n=301(26%) WMH, n=201(17%) CSF Aβ42 and n=199(17%) CSF p-tau. Imputation of these variables were performed on the basis of available data of the variables: age, sex, diagnosis, Mini Mental State Examination (MMSE), Charlson Comorbidity Index (CCI), level of education and APOE genotype. We imputed the variables five times and calculated the mean values.

1. van Buuren S, Boshuizen HC, Knook DL. Multiple imputation of missing blood pressure covariates in survival analysis. Statistics in medicine. 1999;18(6):681-94.
