# Supplementary material for: Development of multivariable prediction models for institutionalization and mortality in the full spectrum of Alzheimer’s disease
Source: Alzheimers Res Ther. 2022 Aug 5;14:110. doi: 10.1186/s13195-022-01053-0 (PMC9354423; doi:10.1186/s13195-022-01053-0)
Supplement: Supplementary file 2 — Additional file 2. Five-fold cross-validation of the prediction models in SCD/MCI patients. [file 13195_2022_1053_MOESM2_ESM.docx]

**Additional file 2. Five-fold cross-validation of the prediction models in SCD/MCI patients**

|  | **Institutionalization** |  |  | **Mortality** |  |  |
| --- | --- | --- | --- | --- | --- | --- |
|  | **Model 2** | **Without CSF** | **Without CSF/MRI** | **Model 2** | **Without CSF** | **Without CSF/MRI** |
| **Age** | 1.03  (0.99; 1.08) | 1.07  (1.02; 1.12) | 1.09  (1.05; 1.14) | 1.04  (1.00; 1.08) | 1.06  (1.02; 1.11) | 1.08  (1.04; 1.13) |
| **Sex, female** | 1.42  (0.79; 2.59) | 1.60  (0.89; 2.86) | 1.46  (0.83; 2.61) | 0.95  (0.61; 1.49) | 0.99  (0.63; 1.54) | 0.86  (0.55; 1.35) |
| **MMSE** | 0.89  (0.79; 0.99) | 0.84  (0.76; 0.93) | 0.84  (0.76; 0.92) |  |  |  |
| **NPI** | 1.04  (1.01; 1.07) | 1.05  (1.01; 1.08) | 1.04  (1.01; 1.07) | 1.03  (1.00; 1.05) | 1.03  (1.00; 1.05) | 1.02  (1.00; 1.05) |
| **CCI** |  |  |  | 1.27  (1.11; 1.46) | 1.23  (1.07; 1.42) | 1.28  (1.13; 1.48) |
| **APOE e4** | 1.80  (0.97; 3.35) | 2.14  (1.19; 3.90) | 1.93  (1.01; 1.07) |  |  | 1.45  (0.96; 2.18) |
| **GCA** | 2.16  (1.34; 3.53) | 2.25  (1.40; 3.60) |  | 1.70  (1.15; 2.51) | 1.68  (1.14; 2.48) |  |
| **MTA** |  |  |  | 1.58  (1.16; 2.16) | 1.57  (1.15; 2.12) |  |
| **WMH** | 1.49  (1.02; 2.18) |  |  |  |  |  |
| **CSF Aβ_42_†** | 0.89  (0.83; 0.95) |  |  | 0.95  (0.91; 0.99) |  |  |
| **CSF p-tau** | 1.03  (1.01; 1.04) |  |  | 1.02  (1.01; 1.04) |  |  |
| **Harrell’s C** | 0.76  (0.64; 0.87) | 0.72  (0.58; 0.86) | 0.73  (0.60; 0.88) | 0.75  (0.66; 0.85) | 0.76  (0.66; 0.86) | 0.74  (0.64; 0.84) |

Data is represented as Hazard Ratio (95%CI) and Harrell’s C (95%CI).

We used all variables as continuous variables in the models, except for the dichotomous variables gender and APOE e4

†Hazard ratio for every 100pg/ml

AD=Alzheimer’s disease, 95%CI= 95% confidence interval, NPI=Neuropsychiatric Inventory, MMSE=mini-mental state examination, CCI=charlson comorbidity index, GCA=global cortical atrophy, MTA=medial temporal lobe atrophy, WMH=white matter hyperintensities, CSF=cerebrospinal fluid, Aβ_42_=β-Amyloid 1–42, p-tau=Tau phosphorylated at threonine 181
