# Supplementary material for: Development of multivariable prediction models for institutionalization and mortality in the full spectrum of Alzheimer’s disease
Source: Alzheimers Res Ther. 2022 Aug 5;14:110. doi: 10.1186/s13195-022-01053-0 (PMC9354423; doi:10.1186/s13195-022-01053-0)
Supplement: Supplementary file 3 — Additional file 3. Five-fold cross-validation of the prediction models in AD dementia. [file 13195_2022_1053_MOESM3_ESM.docx]

**Additional file 3. Five-fold cross-validation of the prediction models in AD dementia**

|  | **Institutionalization** |  |  | **Mortality** |  |  |
| --- | --- | --- | --- | --- | --- | --- |
|  | **Model 2** | **Without CSF** | **Without CSF/MRI** | **Model 2** | **Without CSF** | **Without CSF/MRI** |
| **Age** | 1.00  (0.98; 1.02) | 1.01  (0.99; 1.03) | 1.01  (1.00; 1.03) | 1.02  (1.02; 1.04) | 1.02  (1.00; 1.04) | 1.03  (1.01; 1.04) |
| **Sex. female** | 0.93  (0.75; 1.15) | 0.93  (1.34; 1.16) | 0.89  (0.72; 1.11) | 0.70  (0.56; 0.87) | 0.70  (0.57; 0.88) | 0.71  (0.57; 0.88) |
| **MMSE** | 0.93  (0.91; 0.96) | 0.93  (0.91; 0.95) | 0.92  (0.90; 0.94) | 0.93  (0.91; 0.95) | 0.93  (0.91; 0.95) | 0.93  (0.90; 0.94) |
| **NPI** | 1.03  (1.02; 1.04) | 1.03  (1.02; 1.04) | 1.03  (1.02; 1.04) | 1.01  (1.00; 1.03) | 1.01  (1.00; 1.03) | 1.02  (1.00; 1.03) |
| **CCI** | 0.90  (0.79; 1.01) | 0.90  (0.79; 1.01) | 0.98  (0.79; 0.99) |  |  | 1.11  (0.99; 1.23) |
| **APOE e4** |  |  | 1.23  (0.98; 1.55) |  |  |  |
| **GCA** | 0.19  (0.97; 1.43) | 1.20  (0.98; 1.45) |  | 1.27  (1.03; 1.55) | 1.26  (1.03; 1.55) |  |
| **MTA** | 1.31  (1.11; 1.54) | 1.27  (1.07; 1.49) |  | 1.19  (1.01; 1.39) | 1.17  (1.00; 1.38) |  |
| **WMH** | 0.80  (0.44; 0.94) | 0.79  (0.68; 0.93) |  | 1.20  (1.03; 1.40) | 1.21  (1.03; 1.42) |  |
| **CSF Aβ_42_†** | 1.03  (0.99; 2.07) |  |  | 0.96  (0.92; 1.00) |  |  |
| **CSF p-tau** | 1.01  (1.00; 1.01) |  |  | 1.01  (1.00; 1.01) |  |  |
| **Harrell’s C** | 0.66  (0.59; 0.72) | 0.66  (0.60; 0.72) | 0.66  (0.60; 0.73) | 0.63  (0.56; 0.70) | 0.64  (0.57; 0.71) | 0.64  (0.57; 0.71) |

Data is represented as Hazard Ratio (95%CI) and Harrell’s C (95%CI).

We used all variables as continuous variables in the models, except for the dichotomous variables gender and APOE e4

†Hazard ratio for every 100pg/ml

AD=Alzheimer’s disease, 95%CI= 95% confidence interval, NPI=Neuropsychiatric Inventory, MMSE=mini-mental state examination, CCI=charlson comorbidity index, GCA=global cortical atrophy, MTA=medial temporal lobe atrophy, WMH=white matter hyperintensities, CSF=cerebrospinal fluid, Aβ_42_=β-Amyloid 1–42, p-tau=Tau phosphorylated at threonine 181
