# Supplementary material for: Development of multivariable prediction models for institutionalization and mortality in the full spectrum of Alzheimer’s disease
Source: Alzheimers Res Ther. 2022 Aug 5;14:110. doi: 10.1186/s13195-022-01053-0 (PMC9354423; doi:10.1186/s13195-022-01053-0)
Supplement: Supplementary file 4 — Additional file 4. Schoenfeld residuals for the model (model 2) predicting institutionalization in AD dementia. [file 13195_2022_1053_MOESM4_ESM.docx]

**Additional file 4. Schoenfeld residuals for the model (model 2) predicting institutionalization in AD dementia**

| **Covariates** | **P-value** |
| --- | --- |
| Age | 0.1107 |
| Sex, female | 0.0071 |
| MMSE | 0.0011 |
| NPI | <0.001 |
| MTA | 0.4347 |
| WMH | 0.3009 |
| CSF p-tau | 0.2003 |
| **Global test** | <0.001 |

AD=Alzheimer’s disease, 95%CI= 95% confidence interval, NPI=Neuropsychiatric Inventory, MMSE=mini-mental state examination, MTA=medial temporal lobe atrophy, WMH=white matter hyperintensities, CSF=cerebrospinal fluid, p-tau=Tau phosphorylated at threonine 181
