# Supplementary material for: Development of multivariable prediction models for institutionalization and mortality in the full spectrum of Alzheimer’s disease
Source: Alzheimers Res Ther. 2022 Aug 5;14:110. doi: 10.1186/s13195-022-01053-0 (PMC9354423; doi:10.1186/s13195-022-01053-0)
Supplement: Supplementary file 5 — Additional file 5. Time-varying hazard ratios of MMSE and NPI after six years of follow-up for the model (model 2) predicting institutionalization in AD dementia. [file 13195_2022_1053_MOESM5_ESM.docx]

**Additional file 5. Time-varying hazard ratios of MMSE and NPI after six years of follow-up for the model (model 2) predicting institutionalization in AD dementia**

|  | **Hazard ratio (95%CI)** |
| --- | --- |
| **Main model** |  |
| Age | 0.99 (0.98; 1.01) |
| Sex, female | 0.94 (0.77; 1.14) |
| MMSE | 0.93 (0.92; 0.95) |
| NPI | 1.03 (1.02; 1.04) |
| MTA | 1.32 (1.14; 1.54) |
| WMH | 0.84 (0.73; 0.97) |
| CSF ptau | 1.01 (1.00; 1.01) |
| **Time-varying hazard ratios (>6 years follow-up)** |  |
| MMSE | 1.13 (1.03; 1.23) |
| NPI | 0.97 (0.93; 1.02) |

AD=Alzheimer’s disease, 95%CI= 95% confidence interval, NPI=Neuropsychiatric Inventory, MMSE=mini-mental state examination, CCI=charlson comorbidity index, GCA=global cortical atrophy, MTA=medial temporal lobe atrophy, WMH=white matter hyperintensities, CSF=cerebrospinal fluid, Aβ_42_=β-Amyloid 1–42, p-tau=Tau phosphorylated at threonine 181
