# Supplementary material for: Development of multivariable prediction models for institutionalization and mortality in the full spectrum of Alzheimer’s disease
Source: Alzheimers Res Ther. 2022 Aug 5;14:110. doi: 10.1186/s13195-022-01053-0 (PMC9354423; doi:10.1186/s13195-022-01053-0)
Supplement: Supplementary file 6 — Additional file 6. Univariable and multivariable cox regression models for the prediction of institutionalization and mortality in SCD/MCI amyloid-positive patients. [file 13195_2022_1053_MOESM6_ESM.docx]

**Additional file 6. Univariable and multivariable cox regression models for the prediction of institutionalization and mortality in SCD/MCI amyloid-positive patients**

|  | **Institutionalization** | | | | | **Mortality** | | | | |
| --- | --- | --- | --- | --- | --- | --- | --- | --- | --- | --- |
|  | **Univariable** | **Model 1** | **Model 2** | | | **Univariable** | **Model 1** | **Model 2** | | |
|  |  | **Age and sex adjusted** |  | **Without CSF** | **Without CSF/MRI** |  | **Age and sex adjusted** |  | **Without CSF** | **Without CSF/MRI** |
| **Age** | 1.04  (0.99; 1.09) | 1.04  (0.99; 1.10) | 1.00  (0.94; 1.07) | 1.02  (0.96; 1.09) | 1.04  (0.98; 1.09) | 1.12  (1.06; 1.17) | 1.12  (1.06; 1.17) | 1.07  (1.02; 1.13) | 1.09  (1.03; 1.15) | 1.11  (1.06; 1.17) |
| **Sex, female** | 1.22  (0.61; 2.43) | 1.37  (0.68; 2.79) | 1.19  (0.56; 2.54) | 1.30  (0.62; 2.73) | 1.46  (0.71; 2.98) | 0.59  (0.31; 1.14) | 0.66  (0.35; 1.27) | 0.66  (0.34; 1.30) | 0.73  (0.38; 1.42) | 0.66  (0.35; 1.27) |
| **MMSE** | 0.84*  (0.75; 0.95) | 0.84*  (0.74; 0.95) | 0.84  (0.76; 0.97) | 0.84  (0.74; 0.94) | 0.84  (0.74; 0.95) | 0.93  (0.83; 1.04) | 0.96  (0.86; 1.08) |  |  |  |
| **NPI** | 1.01  (0.96; 1.05) | 1.01  (0.96; 1.06) |  |  |  | 1.03  (0.99; 1.06) | 1.01  (0.97; 1.05) |  |  |  |
| **CCI** | 1.23  (0.95; 1.59) | 1.16  (0.83; 1.63) |  |  |  | 1.58*  (1.28; 1.96) | 1.22  (0.91; 1.66) |  |  |  |
| **APOE e4** | 1.35  (0.61; 2.99) | 1.37  (0.61; 3.04) |  |  |  | 1.01  (0.52; 1.98) | 1.12  (0.57; 2.21) |  |  |  |
| **GCA** | 1.15*  (1.04; 1.27) | 1.15*  (1.02; 1.29) | 1.17  (1.03; 1.34) | 1.19  (1.04; 1.35) |  | 1.20*  (1.09; 1.31) | 1.11  (1.00; 1.23) |  | 1.11  (1.00; 1.23) |  |
| **MTA** | 1.02  (0.61; 1.69) | 0.86  (0.47; 1.56) | 0.57  (0.30; 1.08) | 0.52  (0.27; 0.99) |  | 2.21*  (1.48; 3.31) | 1.55  (0.99; 2.43) | 1.66  (1.04; 2.65) |  |  |
| **WMH** | 1.53*  (1.03; 2.27) | 1.43  (0.94; 2.19) | 1.75  (1.09; 2.81) | 1.66  (1.03; 2.67) |  | 1.31  (0.91; 1.87) | 1.06  (0.71; 1.58) |  |  |  |
| **CSF Aβ_42_†** | 0.90  (0.75; 1.08) | 0.89  (0.74; 1.07) |  |  |  | 0.96  (0.83; 1.12) | 0.97  (0.83; 1.14) |  |  |  |
| **CSF p-tau** | 1.03*  (1.01; 1.05) | 1.03*  (1.01; 1.04) | 1.02  (1.00; 1.04) |  |  | 1.03*  (1.01; 1.04) | 1.02*  (1.00; 1.04) | 1.02  (1.01; 1.04) |  |  |
| **Harrell’s C**  **(95%CI)** |  |  | 0.72  (0.66; 0.84) | 0.73  (0.65; 0.81) | 0.67  (0.59; 0.75) |  |  | 0.74  (0.66; 0.83) | 0.74  (0.65; 0.82) | 0.71  (0.61; 0.81) |

Data is represented as Hazard Ratio (95%CI) and Harrell’s C (95%CI).

We used all variables as continuous variables in the models, except for the dichotomous variables gender and APOE e4

*p<0.05 in univariate analysis

†Hazard ratio for every 100pg/ml

AD=Alzheimer’s disease, 95%CI= 95% confidence interval, NPI=Neuropsychiatric Inventory, MMSE=mini-mental state examination, CCI=charlson comorbidity index, GCA=global cortical atrophy, MTA=medial temporal lobe atrophy, WMH=white matter hyperintensities, CSF=cerebrospinal fluid, Aβ_42_=β-Amyloid 1–42, p-tau=Tau phosphorylated at threonine 181
