# Supplementary material for: Development of multivariable prediction models for institutionalization and mortality in the full spectrum of Alzheimer’s disease
Source: Alzheimers Res Ther. 2022 Aug 5;14:110. doi: 10.1186/s13195-022-01053-0 (PMC9354423; doi:10.1186/s13195-022-01053-0)
Supplement: Supplementary file 7 — Additional file 7. Univariable and multivariable cox regression models for the prediction of institutionalization and mortality in amyloid-positive patients with AD dementia. [file 13195_2022_1053_MOESM7_ESM.docx]

**Additional file 7. Univariable and multivariable cox regression models for the prediction of institutionalization and mortality in amyloid-positive patients with AD dementia**

|  | **Institutionalization** | | | | | **Mortality** | | | | |
| --- | --- | --- | --- | --- | --- | --- | --- | --- | --- | --- |
|  | **Univariable** | **Model 1** | **Model 2** | | | **Univariable** | **Model 1** | **Model 2** | | |
|  |  | **Age and sex adjusted** |  | **Without CSF** | **Without CSF/MRI** |  | **Age and sex adjusted** |  | **Without CSF** | **Without CSF/MRI** |
| **Age** | 1.00  (0.99; 1.02) | 1.00  (0.99; 1.02) | 1.00  (0.98; 1.01) | 1.00  (0.98; 1.02) | 1.00  (0.99; 1.02) | 1.02*  (1.00; 1.04) | 1.02*  (1.00; 1.03) | 1.01  (0.99; 1.02) | 1.01  (0.99; 1.02) | 1.02  (1.01; 1.04) |
| **Sex, female** | 0.94  (0.77; 1.16) | 0.94  (0.77; 1.16) | 0.90  (0.73; 1.12) | 0.91  (0.74; 1.13) | 0.97  (0.71; 1.08) | 0.74*  (0.60; 0.91) | 0.74*  (0.60; 0.92) | 0.68  (0.55; 0.85) | 0.68  (0.55; 0.85) | 0.70  (0.57; 0.86) |
| **MMSE** | 0.93*  (0.91; 0.94) | 0.92*  (0.91; 0.94) | 0.94  (0.92; 0.96) | 0.93  (0.92; 0.95) | 0.93  (0.91; 0.95) | 0.93*  (0.91; 0.95) | 0.93*  (0.91; 0.95) | 0.94  (0.92; 0.96) | 0.94  (0.92; 0.96) | 0.93  (0.91; 0.95) |
| **NPI** | 1.04*  (1.03; 1.05) | 1.04*  (1.03; 1.05) | 1.03  (1.02; 1.04) | 1.03  (1.02; 1.04) | 1.03  (1.02; 1.04) | 1.02*  (1.01; 1.03) | 1.02*  (1.01; 1.03) | 1.01  (1.00; 1.03) | 1.01  (1.00; 1.03) | 1.01  (1.00; 1.03) |
| **CCI** | 1.00  (0.92; 1.09) | 1.00  (0.89; 1.12) |  |  |  | 1.13*  (1.04; 1.23) | 1.08  (0.97; 1.21) |  |  |  |
| **APOE e4** | 1.04  (0.83; 1.31) | 1.04  (0.83; 1.30) |  |  |  | 0.88  (0.71; 1.01) | 0.87  (0.69; 1.08) |  |  |  |
| **GCA** | 1.04*  (1.00; 1.07) | 1.04*  (1.00; 1.08) |  |  |  | 1.07*  (1.03; 1.11) | 1.06*  (1.02; 1.10) |  |  |  |
| **MTA** | 1.38*  (1.19; 1.60) | 1.44*  (1.23; 1.69) | 1.36  (1.15; 1.60) | 1.32  (1.12; 1.55) |  | 1.42*  (1.24; 1.63) | 1.37*  (1.18; 1.58) | 1.14  (0.98; 1.34) | 1.14  (0.98; 1.34) |  |
| **WMH** | 0.96  (0.83; 1.10) | 0.95  (0.82; 1.11) | 0.83  (0.71; 0.97) | 0.82  (0.70; 0.96) |  | 1.38*  (1.19; 1.58) | 1.37*  (1.18; 1.59) | 1.27  (1.09; 1.48) | 1.27  (1.09; 1.48) |  |
| **CSF Aβ_42_†** | 0.95  (0.89; 1.01) | 0.95  (0.89; 1.01) |  |  |  | 0.96  (0.90; 1.02) | 0.97  (0.91; 1.03) |  |  |  |
| **CSF p-tau** | 1.01*  (1.00; 1.01) | 1.01*  (1.00; 1.01) | 1.01  (1.00; 1.01) |  |  | 1.01  (1.00; 1.01) | 1.01  (1.00; 1.01) |  |  |  |
| **Harrell’s C**  **(95%CI)** |  |  | 0.68  (0.64; 0.71) | 0.68  (0.64; 0.71) | 0.67  (0.64; 0.70) |  |  | 0.65  (0.62; 0.68) | 0.65  (0.62; 0.68) | 0.64  (0.62; 0.68) |

Data is represented as Hazard Ratio (95%CI) and Harrell’s C (95%CI).

We used all variables as continuous variables in the models, except for the dichotomous variables gender and APOE e4

*p<0.05 in univariate analysis

†Hazard ratio for every 100pg/ml

AD=Alzheimer’s disease, 95%CI= 95% confidence interval, NPI=Neuropsychiatric Inventory, MMSE=mini-mental state examination, CCI=charlson comorbidity index, GCA=global cortical atrophy, MTA=medial temporal lobe atrophy, WMH=white matter hyperintensities, CSF=cerebrospinal fluid, Aβ_42_=β-Amyloid 1–42, p-tau=Tau phosphorylated at threonine 181
