# Supplementary material for: Development of multivariable prediction models for institutionalization and mortality in the full spectrum of Alzheimer’s disease
Source: Alzheimers Res Ther. 2022 Aug 5;14:110. doi: 10.1186/s13195-022-01053-0 (PMC9354423; doi:10.1186/s13195-022-01053-0)
Supplement: Supplementary file 8 — Additional file 8. Baseline characteristics Memento cohort. [file 13195_2022_1053_MOESM8_ESM.docx]

**Additional file 8. Baseline characteristics Memento cohort**

|  | **SCD/MCI (n=2308)** |
| --- | --- |
| **Age** | 71±9 |
| **Female** | 1430 (62%) |
| **MMSE** | 28±2 |
| **Diagnosis** |  |
| **SCD** | 365 (16%) |
| **MCI** | 1943 (84%) |
| **NPI** | 2.6±6.0 |
| **CCI** | 3.1±1.3 |
| **APOE e4 carrier** | 651 (30%) |
| **MRI** |  |
| **Brain parenchymal fraction** | 73±5% |
| **MTA** | 1.3±0.8 |
| **WMH** | 1.2±0.8 |
| **CSF, pg/ml** |  |
| **Aβ42** | 1652±761 |
| **p-tau** | 32±14 |

Data is represented as mean±SD, median (range) or n(%).

SCD= subjective cognitive decline, MCI= mild cognitive impairment; MMSE=Mini-Mental State examination; NPI= Neuropsychiatric Inventory; CCI= Charlson Comorbidity Index; MRI= magnetic resonance imaging; GCA= Global cortical atrophy; MTA= Medial temporal lobe atrophy; WMH= white matter hyperintensities, CSF=cerebrospinal fluid, Aβ42=β-Amyloid 1–42.
