# Supplementary material for: Development of multivariable prediction models for institutionalization and mortality in the full spectrum of Alzheimer’s disease
Source: Alzheimers Res Ther. 2022 Aug 5;14:110. doi: 10.1186/s13195-022-01053-0 (PMC9354423; doi:10.1186/s13195-022-01053-0)
Supplement: Supplementary file 9 — Additional file 9. Harrell’s C Memento cohort based on regression variables of the Amsterdam Dementia Cohort (ADC) models. [file 13195_2022_1053_MOESM9_ESM.docx]

**Additional file 9. Harrell’s C Memento cohort based on regression variables of the Amsterdam Dementia Cohort (ADC) models**

|  | **SCD/MCI Memento cohort (n=2308)** | | | | | |
| --- | --- | --- | --- | --- | --- | --- |
|  | **Institutionalization** | | | **Mortality** | | |
|  |  | **Without CSF** | **Without CSF/MRI** |  | **Without CSF** | **Without CSF/MRI** |
| **Harrell’s C**  **(95%CI)** | 0.79  (0.65; 0.93) | 0.76  (0.70; 0.81) | 0.79  (0.74; 0.85) | 0.72  (0.60; 0.85) | 0.73  (0.68; 0.79) | 0.73  (0.68; 0.78) |

95%CI= 95% confidence interval, CSF=cerebrospinal fluid
